# Safety and efficacy of PD-1 inhibitors plus tyrosine kinase inhibitors combination therapy in patients with advanced hepatocellular carcinoma combined with hyperbilirubinemia: a retrospective cohort study

**DOI:** 10.3389/fimmu.2025.1530477

**Published:** 2025-03-11

**Authors:** Shida Pan, Jianing Wang, Jiahe Tian, Yilin Wang, Siyu Wang, Yingying Yu, Fengyi Li, Yan-Mei Jiao, Yingjuan Shen, Luo Yang, Xiaomeng Liu, Qin Qiu, Junqing Luan, Fu-Sheng Wang, Fanping Meng

**Affiliations:** ^1^ Beijing Ditan Hospital, Capital Medical University, Beijing, China; ^2^ Senior Department of Infectious Diseases, The Fifth Medical Center of Chinese People's Liberation Army (PLA) General Hospital, Beijing, China; ^3^ Peking University 302 Clinical Medical School, Beijing, China; ^4^ Chinese People's Liberation Army (PLA) Medical School, Beijing, China; ^5^ The First Affiliated Hospital of University of Science and Technology of China (USTC), Division of Life Sciences and Medicine, University of Science and Technology of China, Hefei, China

**Keywords:** hepatocellular carcinoma, hyperbilirubinemia, programmed death 1, immunotherapy, survival

## Abstract

**Background:**

Programmed death-1 (PD-1) inhibitors plus tyrosine kinase inhibitors (TKIs) combination therapy are considered as a first-line treatment recommendation for advanced hepatocellular carcinoma (HCC). However, patients with hyperbilirubinemia are excluded from this therapeutic option due to limitations in indications. There is a notable absence of published studies evaluating the safety and efficacy of the PD-1 inhibitors plus TKIs combination therapy in patients with HCC combined with hyperbilirubinemia.

**Methods:**

Patients with HCC complicated with hyperbilirubinemia who received combination therapy with PD-1 inhibitors and TKIs were retrospectively analyzed. Adverse events, tumor response, and laboratory parameters were recorded to assess the safety and efficacy of the treatment, as well as to identify potential risk factors influencing survival.

**Results:**

A total of 108 participants were included in the study, with 56 patients (51.9%) reporting at least one adverse event, the majority of which were mild. The objective response rate (ORR) for the enrolled participants was 11.9%, and the disease control rate(DCR) reached 61.2%. The median overall survival (OS) for the entire cohort was 5.03 months, while the median progression-free survival (PFS) was 3.63 months. Multifactorial analysis showed that MELD score >18 and increased total bilirubin (TBIL) levels within one week were significant risk factors for OS. Patients with a decrease in TBIL levels within one week had significantly prolonged median OS (not reached vs 3.3months, *P* =0.013) and median PFS (7.03 months vs 2.77 months, *P* =0.010).

**Conclusion:**

Combination therapy demonstrated favorable safety and tolerability among patients with HCC combined with hyperbilirubinemia. Patients who experienced a rapid decline in TBIL levels during the early phase of treatment with PD-1 inhibitors and TKIs were observed to derive clinical benefits. Early initiation of aggressive interventions aimed at reducing TBIL levels is recommended to optimize treatment outcomes.

## Introduction

Hepatocellular carcinoma (HCC) is among the most widespread and lethal malignancies globally (1). It is the fifth most common cancer and the third leading cause of cancer-related mortality globally (2, 3). Most patients have lost their chance for radical surgical resection due to the diagnosis of advanced HCC (4), which also results in limited overall survival (OS) and unimproved prognosis (5). For these patients, multiple treatment options, including surgical resection, percutaneous ablation, and radiotherapy, have demonstrated potential benefits. However, there are significant limitations regarding their efficacy and suitability for specific patient populations (6).

Immune checkpoint inhibitors (ICIs) such as programmed death-1/programmed death ligand-1 (PD-1/PD-L1) inhibitors or cytotoxic T lymphocyte associated protein-4 (CTLA-4) inhibitors, combined with tyrosine kinase inhibitors (TKIs), have gained prominence in hepatocellular carcinoma (HCC) study, demonstrating promising therapeutic potential (7, 8). The combination of atezolizumab with bevacizumab currently stands as the preferred first-line therapeutic regimen for advanced HCC, extending the median overall survival (mOS) of patients to 19.2 months, accompanied by an increased objective response rate (ORR) of 27.3% (7). The HIMALAYA phase III clinical study demonstrated a 22% reduction in the risk of death for patients treated with tremelimumab plus durvalumab compared to sorafenib (8).

However, PD-1 inhibitors plus TKIs combination therapy is limited by liver function due to concerns that adverse events (AEs) may lead to liver failure (8). According to instructions for PD-1 inhibitors (9–11) and NCCN clinical practice guidelines (12), patients with moderate to severe liver injury or combined with hyperbilirubinemia are not recommended to be treated with ICIs in combination with TKIs. Patients with elevated total bilirubin (TBIL) have been considered unsuitable for combination therapy in the past; hence, previous studies have not enrolled these patients. According to the recommendations of the guidelines, symptomatic treatment such as percutaneous hepatic puncture biliary drainage (PTCD) or bile duct stenting (BA) is usually given priority to improve cholestasis, and immunotherapy is considered after the level of bilirubin decreases (12). However, even if some patients receive the above intervention, TBIL is still difficult to effectively fall back to the safe threshold. There is no standard treatment for this particular group of people. However, these patients exhibit a considerable potential demand for novel therapeutic strategies. According to the Asian Pacific Association for the Study of the Liver (APASL) guidelines definition of liver failure, elevated TBIL levels alone do not constitute liver failure (13). TBIL levels exceeding the upper limit of normal (ULN) with INR <1.5 and/or prothrombin activity (PTA) ≥40%, indicate the presence of simple cholestasis rather than of liver failure (13). Previous studies have often used surgical treatments to rapidly lower TBIL levels before initiating PD-1 combined with TKIs therapy, which greatly limits the scope of application and timing of combination therapy (14). Limited research has extensively investigated the safety and efficacy of the PD-1 inhibitors plus TKIs combination therapy in patients with advanced unresectable HCC combined with hyperbilirubinemia, especially patients with TBIL level >3×ULN who are not recommended for combination therapy.

In this retrospective study, we investigated the efficacy and safety of PD-1 inhibitors plus TKIs combination therapy in patients with HCC combined with hyperbilirubinemia and analyzed the risk factors for survival.

## Materials and methods

### Participants

We conducted a retrospective analysis of 230 HCC patients who received combination therapy with PD-1 inhibitors and TKIs at the Department of Biological Injury, Fifth Medical Center of PLA General Hospital between June 2020 and August 2024. Among these patients, we further selected those with baseline TBIL levels >3×ULN and PTA ≥40% for subsequent analysis (n=108). Hyperbilirubinemia is defined as bilirubin levels exceeding the ULN (ULN = 17.1 μmol/L) (15, 16). These patients were not recommended for treatment with ICIs, according to the prescribing information for PD-1 inhibitors and the NCCN clinical practice guidelines (12, 17, 18). These patients were refractory to standard interventions, including PTCD and BA therapy. Prior to initiating combination therapy with ICIs and TKIs, these patients were managed solely with conservative supportive care. Given the absence of alternative therapeutic options and after thorough multidisciplinary team evaluation, we proceeded with PD-1 plus TKIs combination therapy in this patient cohort. Comprehensive safety monitoring and efficacy assessments were implemented. The patient inclusion criteria were as follows: age ≥18 years; histologically/imaging confirmed advanced HCC or intrahepatic cholangiocarcinoma (ICC); patients who received at least one cycle of ICIs combined with TKIs; TBIL level at baseline >3× ULN (51 μmol/L); PTA at baseline >40%; and at least one measurable target lesion based on modified Response Evaluation Criteria in Solid Tumors (mRECIST) (19, 20). The exclusion criteria were as follows: combination of other primary tumors; severe coagulation disorders; combination of severe cardiac and renal insufficiency; and other diseases such as diabetes and hypertension that were under control. The study was approved by the Ethics Committee of The Fifth Medical Center of the Chinese PLA General Hospital (IRB No. KY-2023-7-46-1), with informed consent waived due to its retrospective nature.

### Drug administration

The PD-1 inhibitors: sintilimab (Innovent Biologics, China), camrelizumab (Hengrui Pharma, China), and tislelizumab (BeiGene, China) were prescribed at a fixed dose of 200 mg every three weeks, while toripalimab (Top Alliance, China) was administered at 240 mg. The PD-1/CTLA-4 bispecific inhibitor, cadonilimab (Akesobio, China), were prescribed at 240 mg every three weeks. Sorafenib (Bayer Schering Pharma, Germany) was administered daily at 400 mg, lenvatinib (Eisai, Japan) at 8 to 12 mg/day according to body weight, and bevacizumab (Qilu Pharmaceutical, China) at 7.5 mg/kg every three weeks. The choice of agents was based on physician discretion, patient tolerance, and individual financial considerations of patients. Treatment options for patients who met the criteria for transcatheter arterial chemoembolization (TACE), PTCD or BA treatment were decided together by the patient and the physician before or after systemic combination therapies. Among the enrolled patients, 65 patients were identified with confirmed history of HBV infection and continued to receive NAs during the treatment to control viral replication. NAs including entecavir (ETV) (0.5 mg/day), tenofovir (TDF) (300 mg/day), and adefovir dipivoxil (ADV) (10 mg/day) were administered during immunotherapy.

### Tumor response and adverse event evaluation

Baseline radiological responses were documented using computed tomography (CT) or magnetic resonance imaging (MRI), with subsequent assessments performed every three months. Tumor responses were evaluated by mRECIST (19, 20). Complete response (CR) was defined as the disappearance of arterial enhancement in all target lesions based on the sum of baseline diameters; partial response (PR) was designated when the sum of target lesion diameters decreased by at least 30%, while an increase of at least 20% in the sum of diameters was defined as progressive disease (PD). Stable disease (SD) was characterized by no change in the sum of diameters between PR and PD. AEs were recorded according to the Common Terminology Criteria for Adverse Events (CTCAE) version 5.0 (17, 21). Patients who experience AEs associated with ICIs or TKIs therapy may undergo dose reduction, treatment suspension, discontinuation, or a switch to alternative agents depending on the severity of toxicity. Immunosuppression was administered according to the severity of the AEs.

### Measurement

The cutoff date for follow-up was August 31, 2024, with data extracted from patient medical records. The measurements of the study were similar to those of a previous study (22). Baseline information comprised patient demographic details, such as the Child–Pugh stage, Barcelona Clinic Liver Cancer (BCLC) stage, type of combination therapy, TACE, PTCD and BA treatment. Data collection encompassed baseline, one-week, and three-month routine blood tests, hepatic functional assessments, lymphocyte subset characterization, along with quantification of C-reactive protein (CRP) and interleukin-6 (IL-6). Serum CRP levels and hepatic functional assessments were quantified employing the automated biochemical analyzer (Beckman Coulter AU5400, CA). Serum IL-6 levels were evaluated utilizing the Roche Cobas 8000 system (Roche Diagnostics GmbH, Germany). The lymphocyte subsets were measured from whole blood, and the following subsets were identified: total lymphocytes (CD45+), CD8+ T cells (CD45+CD3+CD8+), CD4+ T cells (CD45+CD3+CD4+), NK cells (CD45+CD3-CD16+CD56+), and B cells (CD45+CD3-CD19+). The cells were stained with the following human antibodies: anti-CD3-FITC, anti-CD45-PerCP, anti-CD8-PE, anti-CD4-APC, anti-CD16-PE, anti-CD56-PE, and anti-CD19-APC. All antibodies were sourced from BD Biosciences (Franklin Lakes, NJ). For the gating strategy, we first identified lymphocytes based on CD45 expression and light scatter properties. Subsequently, T cells were gated as CD3+ cells and further subdivided into CD4+ and CD8+ subsets. NK cells were identified as CD3-CD16+CD56+, and B cells as CD3-CD19+. Routine blood parameters were analyzed using the automated hematology analyzer (SYSMEX HN-2000 series, Japan).

### Statistics

All statistical analyses were conducted using the SPSS Statistics version 27.0 (IBM, USA). Baseline data and adverse events were summarized using descriptive statistics. Continuous variables were presented as mean ± standard deviation(SD) or median (interquartile ranges[IQR]), while categorical variables were presented as counts and percentages. Student t test and nonparametric test were used to compare continuous variables. Categorical variables were compared using Fisher’s exact test or the χ² test. The cutoff date for survival analysis was August 31, 2024. OS and progression-free survival (PFS) were assessed using Kaplan–Meier curves with comparisons facilitated by the log-rank test. Cox survival analysis (Backward-Wald) was used to analyze hazard ratios (HRs) and 95% confidence intervals (CIs), identifying independent risk factors associated with OS. Variables with *P <*0.1 in univariate analysis, as well as those deemed necessary for adjustment by the researchers, were included in the subsequent multivariate analysis. Data were visualized using GraphPad Prism version 9.4 (GraphPad Software, USA). Statistical significance was set at *P <*0.05.

## Results

### Baseline characteristics of patients

In our cohort, 108 participants were included, comprising patients with HCC (n=75, 69.4%) and patients with ICC (n=33, 30.6%) (**Supplementary Figure S1**). Among the patients receiving ICI treatment, 61 patients were treated with sintilimab, 28 patients with cadonilimab, and 19 patients with other PD-1 inhibitors. A total of 86 participants received combination therapy with lenvatinib, and 22 patients received sorafenib, bevacizumab or rigotinib in combination. Among the 38 patients who underwent PTCD treatment during the combination therapy, TBIL levels could not be effectively corrected. Notably, 79% of these patients received PTCD treatment prior to immunotherapy. A total of 71 patients had a history of chronic viral hepatitis (hepatitis B or C) and 65 of them received antiviral interventions. Twenty-five percent of patients underwent TACE within one week prior to immunotherapy. Patients with severe TACE-related AEs (e.g., fever, hemorrhage) were excluded. Among the 108 patients, the median baseline TBIL was 105.50 μmol/L, and the median baseline PTA was 68.60%. The median MELD score was 11.66. Further details are listed in **Table 1**.

**Table 1:** Baseline characteristics of patients with level of TBIL ≥ 3xULN enrolled in the study.

	n=108 (%)
Age (year)	55.81 ± 10.19
Sex	
Male	95 (88.0%)
Female	13 (12.0%)
Diagnosis	
Hepatocellular carcinoma	75 (69.4%)
Cholangiocarcinoma	33 (30.6%)
Basics of hepatitis	
Viral hepatitis B	65 (60.2%)
Viral hepatitis C	6 (5.6%)
Alcoholic hepatitis	15 (13.9%)
Without basics of hepatitis	21 (19.4%)
Other	1 (0.9%)
BCLC stage	
A	2 (2.7%)
B	4 (5.3%)
C (PVTT)	20 (26.7%)
C (M)	26 (34.7%)
D	23 (30.7%)
Tumor node metastasis classification	
III	11 (33.3%)
IV	22 (66.7%)
Child-Pugh stage	
A	5 (4.6%)
B	80 (74.1%)
C	23 (21.3%)
mALBI	
1	5 (4.6%)
2a	4 (3.7%)
2b	39 (36.1%)
3	60 (55.6%)
AFP	
<400 ng/ml	65 (60.2%)
≥400 ng/ml	41 (38.0%)
Not available	2 (1.9%)
CA19-9	
≤37 U/ml	23 (21.3%)
>37 and <1000 U/ml	64 (59.3%)
≥1000 U/ml	10 (9.3%)
Not available	11 (10.2%)
Baseline of TBIL (μmol/L)	105.50 (65.03, 199.60)
Baseline of PA (%)	68.60 (56.90, 80.90)
MELD	11.66 ± 5.39
Previous antiviral therapy	
Yes	65 (60.2%)
No	43 (39.8%)
Immune-related adverse events	
Yes	56 (51.9%)
No	52 (48.1%)
Immunotherapy	
Sintilimab	61 (56.5%)
Camrelizumab	7 (6.5%)
Tislelizumab	7 (6.5%)
Cadonilimab	28 (25.9%)
Toripalimab	4 (3.7%)
Durvalumab	1 (0.9%)
Combination TKI treatment	
Lenvatinib	86 (79.6%)
Bevacizumab	5 (4.6%)
Sorafenib	3 (2.8%)
Rigotinib	13 (12.0%)
Apatinib	1 (0.9%)
Combinated TACE therapy	
Yes	27 (25.0%)
No	81 (75.0%)
Combinated BA therapy	
Yes	7 (6.5%)
No	101 (93.5%)
Combinated PTCD therapy	
Yes (before/after combinational therapy)	[38 (30/8)] (35.2%)
No	70 (64.8%)
Lines of systemic therapy	
First line	80 (74.1%)
Second line	24 (22.2%)
Later line	4 (3.7%)

Continuous variables are presented as mean ± SD or median (interquartile ranges). Immune-related adverse events (irAEs) refer to adverse events that arise from the activation of the immune system due to immune checkpoint inhibitors (such as PD-1/PD-L1 inhibitors). AFP, alpha fetoprotein; BA, Bilirubin adsorption; BCLC, barcelona clinic liver cancer; MELD, model for end-stage liver disease; PA, prothrombin activity; PTCD, percutaneous transhepatic cholangio drainage; TACE, transcatheter arterial chemoembolization TBIL, total bilirubin; TKI, tyrosine kinase inhibitor.

### Safety and tolerability: treatment-related adverse events and liver functions

In this study, the administration of combination therapy demonstrated overall good tolerability. Among the patients, 51.9% (n=56) experienced at least one treatment-related adverse events (TRAEs) of any grade, and 15.7% of patients (n=17) reported grade 3 (G3) or grade 4 (G4) TRAEs. The most common TRAEs reported were fever (n=18, 16.7%), increased TBIL (n=12, 11.1%), rash (n=8, 7.4%), pruritus (n=8, 7.4%) and fatigue (n=7, 6.5%). The most frequent G3/4 TRAEs primarily manifested as increased TBIL and rashes. All patients who experienced severe adverse events (AEs) showed improvement following treatment with corticosteroids. The overview of AEs occurrences in the patient population is provided in **Table 2**. During treatment, sintilimab was permanently discontinued in two patients due to AEs. One patient who experienced AEs suspended sintilimab. Subsequently, this patient was rechallenged with sintilimab after improvement in AEs and did not suffer G3/4 AEs. One patient discontinued camrelizumab due to cutaneous hemangioma formation. Three patients reduced the dose of TKIs due to AEs; two patients discontinued TKIs because of complications while five others switched to regorafenib due to tumor progression. Overall, the incidence and nature of AEs appeared to be generally consistent with those in our previous study cohort (22).

**Table 2:** Occurrence of adverse events.

	G1/2	G3	G4
Fever	18 (16.7%)	0	0
Pruritus	7 (6.5%)	1 (0.9%)	0
Fatigue	7 (6.5%)	0	0
Elevated TBIL	6 (5.6%)	6 (5.6%)	0
Decreased of appetite	5 (4.6%)	2 (1.9%)	0
Rash	4 (3.7%)	3 (2.8%)	1 (0.9%)
Vomiting	3 (2.8%)	1 (0.9%)	0
Abdominal distention	3 (2.8%)	1 (0.9%)	0
Stomachache	2 (1.9%)	2 (1.9%)	0
Diarrhea	2 (1.9%)	0	0

TBIL, total bilirubin.

**Figure 1:**
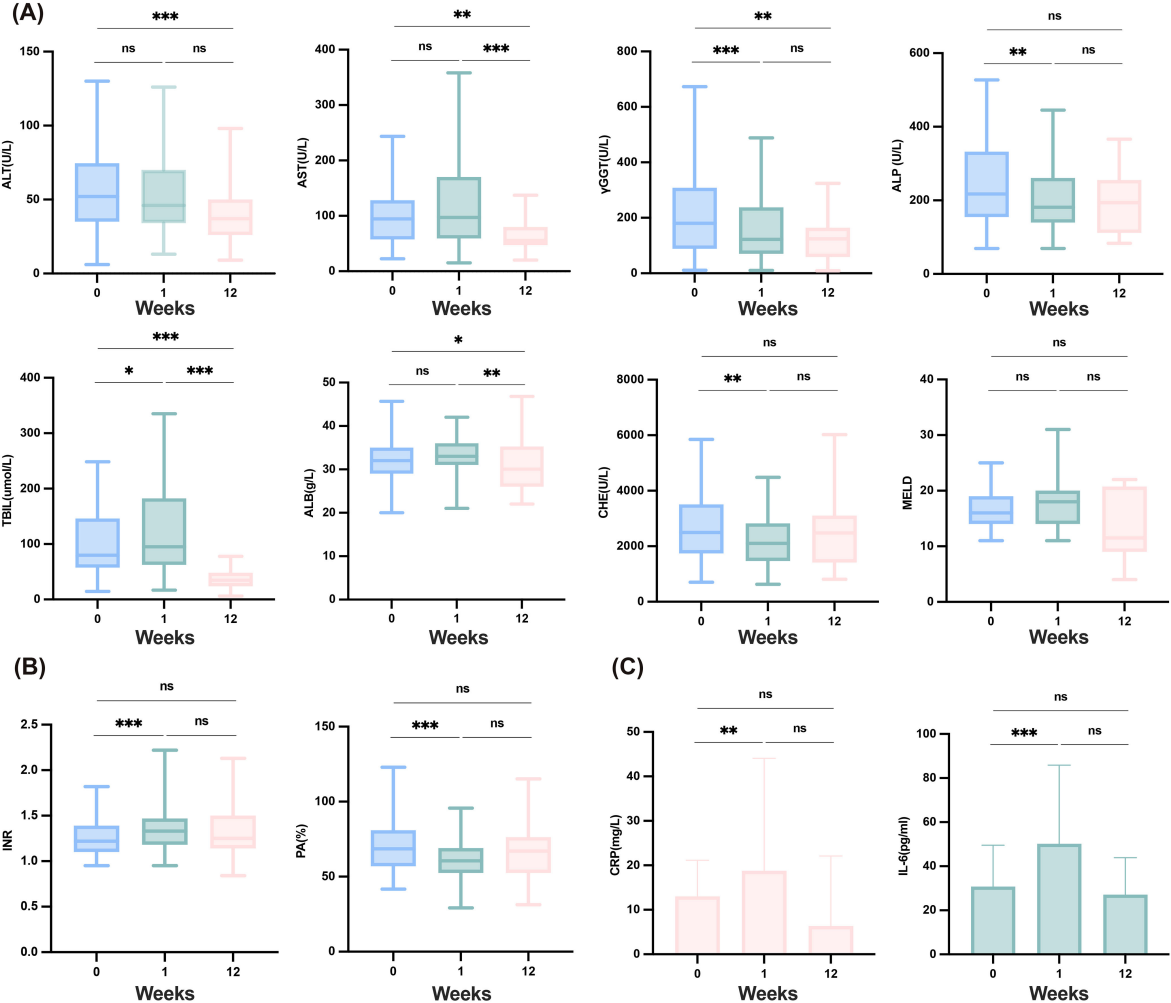
Safety of the combination therapy and changes in liver function, coagulation and inflammatory factors in12 weeks. **(A)** Changes in liver function after combination therapy at baseline, one week and 12weeks. **(B)** Changes in coagulation after combination therapy at baseline, one week and 12weeks. **(C)** Changes in inflammatory factors after combination therapy at baseline, one week and 12weeks. ALB, albumin; ALP, alkaline phosphatase; ALT, alanine aminotransferase; AST, aspartate aminotransferase; CHE, cholinesterase; CRP, C-reactive protein; γGGT, gamma-glutamyl transferase; IL-6, interleukin-6; INR, international normalized ratio; MELD, model for end-stage liver disease; PA, prothrombin activity; TBIL, total bilirubin. ***≤0.005; **≤0.01; *<0.05; ns, ≥0.05.

To evaluate the patient’s tolerance to the combination therapy, we assessed baseline characteristics and tracked early changes in key clinical parameters (**Figure 1A**). Compared with baseline levels, significant improvements in liver inflammation-related markers were observed after 12 weeks of treatment, including the levels of alanine aminotransferase (ALT) (*P <*0.001), Aspartate aminotransferase (AST) (*P* =0.008), gamma-glutamyltransferase (γGT) (*P* =0.003), and TBIL (P<0.001). γGT levels significantly decreased within one week (*P <*0.001). The MELD score did not show a significant increase during the treatment period (*P* =0.742), indicating that the combined therapy did not exacerbate liver burden, and overall liver function remained stable, with early improvements noted in cholestasis-related markers. It is noteworthy that serum albumin (ALB) levels significantly decreased at 12 weeks (*P* =0.021), potentially due to inadequate nutritional intake following patient discharge. Coagulation functions such as PTA and INR did not show significant differences compared to baseline levels after 12 weeks of treatment (*P* =0.197, *P* =0.240); however, an improving trend was observed at 12 weeks (**Figure 1B**). Furthermore, compared with the established baseline level, the level of IL-6 showed a significant increase at one week (*P* < 0.001), but there was no significant difference at 12 weeks relative to baseline level (*P* =0.938). The trend in level of CRP paralleled that of IL-6 (**Figure 1C**).

### Anti-tumor activity: treatment response profiles and immunity

**Figure 2:**
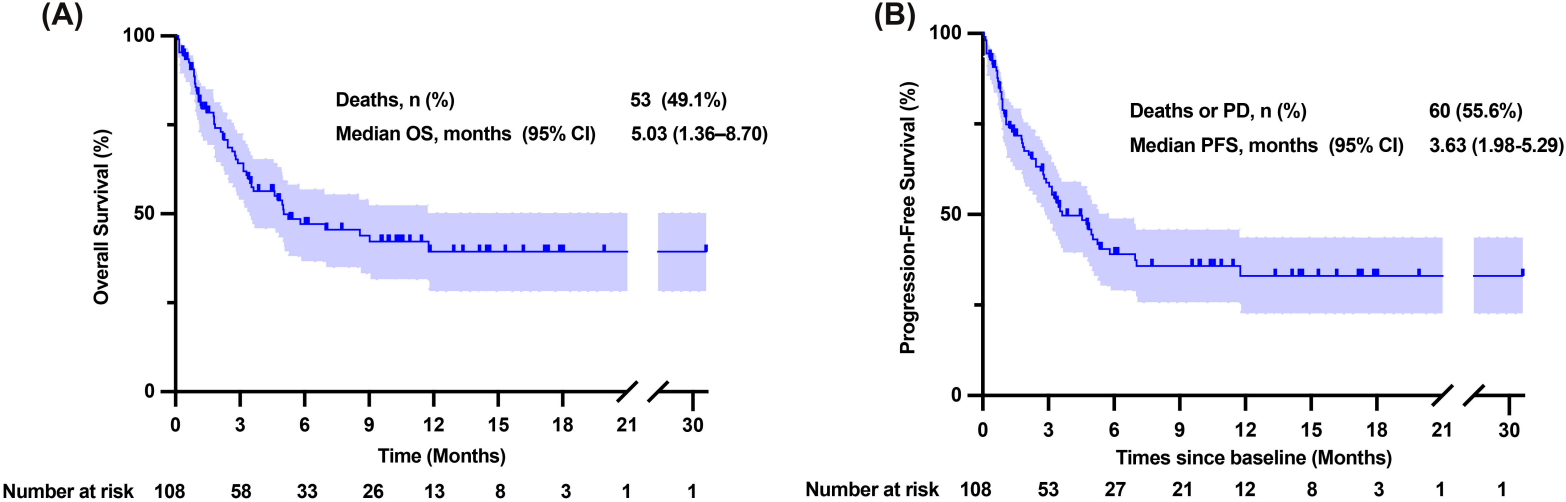
Overall survival and progression-free survival time of patients enrolled. **(A)** Kaplan-Meier curve of overall survival time. **(B)** Kaplan-Meier curve of progression-free survival time. CI, confidence interval; NR, Not reached; OS, overall survival; PD, progressive disease; PFS, progression-free survival.

Tumor responses were assessed in all patients using the mRECIST criteria, as shown in **Table 3**. Among the 67 patients who could be evaluated for tumor response, one patient achieved CR, seven patients achieved PR, 33 patients had SD, and 26 patients had PD. Notably, 10 patients were lost to follow-up during the study. The ORR was 11.9%, while the disease control rate (DCR) reached 61.2%. We analyzed patient survival with a median follow-up of 9.60 months (95% CI: 6.68–12.52 months), in which 53 patients died and ten patients were lost to follow-up. The mOS was 5.03 months (95% CI: 1.36–8.70 months) (**Figure 2A**). Excluding patients lost to follow-up, 60 patients experienced either tumor progression or death, with a median progression-free survival (median PFS) of 3.63 months (95% CI: 1.98–5.29 months) (**Figure 2B**).

**Table 3:** Tumor response profiles.

Tumor response (mRECIST)	n=108 (%)
CR	1 (0.9%)
PR	7 (6.5%)
SD	33 (30.6%)
PD	26 (24.1%)
Unable to evaluate	31 (28.7%)
Loss to follow-up	10 (9.3%)
ORR (CR+PR)	8 (11.9%)
DCR (CR+PR+SD)	41 (61.2%)

CR, complete response; DC, disease control; mRECIST, modified Response Evaluation Criteria in Solid Tumors; OR, objective response; PD, progressive disease; PR, partial response; SD, stable disease.

**Figure 3:**
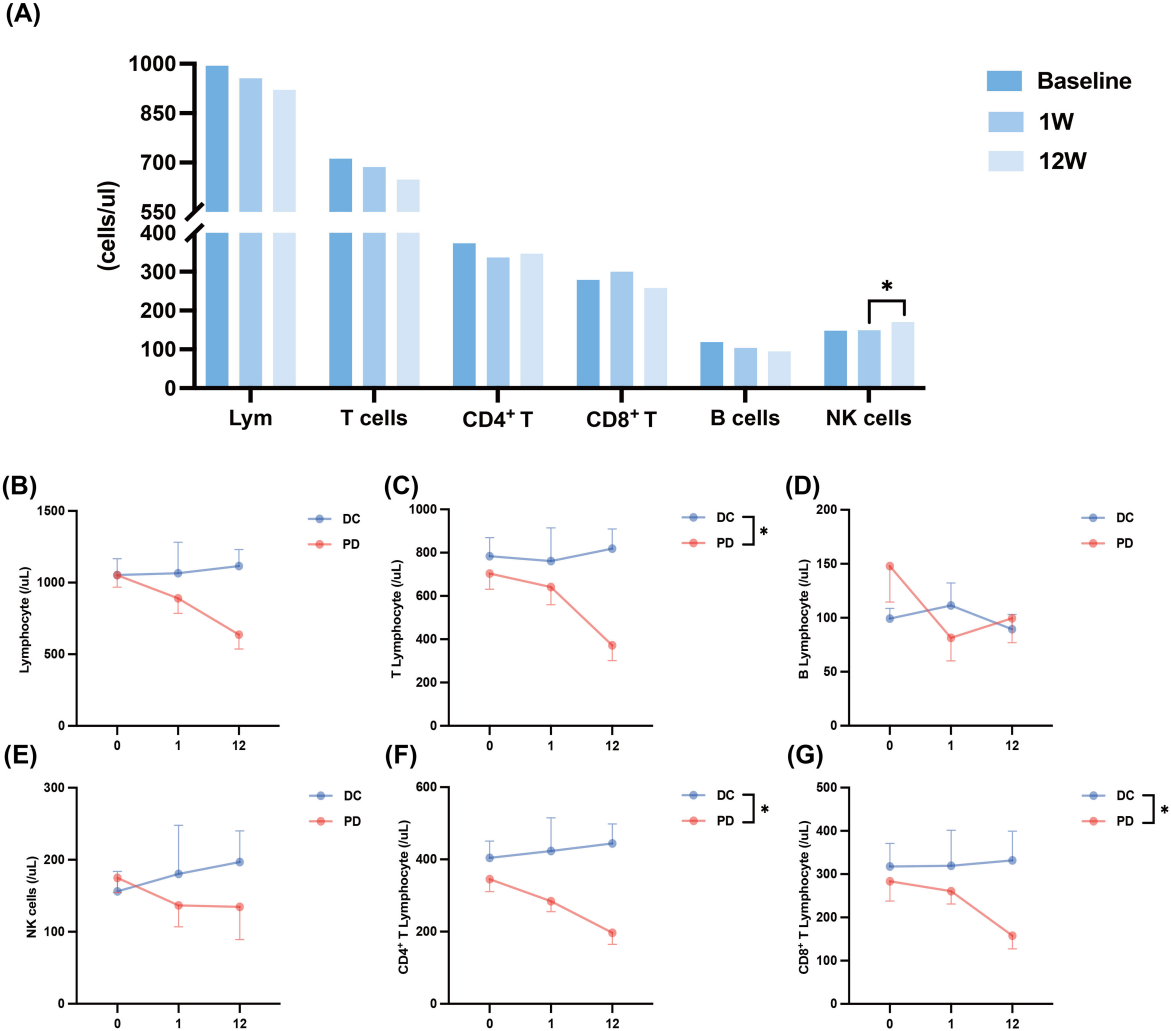
The changes in lymphocyte subsets after combination therapy. **(A)** Changes in lymphocyte subsets after combination therapy at baseline, one week and 12weeks. **(B)** Changes in lymphocyte count within 12 weeks of combination therapy in patients with DC and patients with PD. **(C)** Changes in T cell count within 12 weeks of combination therapy in patients with DC and patients with PD. **(D)** Changes in B cell count within 12 weeks of combination therapy in patients with DC and patients with PD. **(E)** Changes in NK cell count within 12 weeks of combination therapy in patients with DC and patients with PD. **(F)** Changes in CD4^+^ T cell count within 12 weeks of combination therapy in patients with DC and patients with PD. **(G)** Changes in CD8^+^ T cell count within 12 weeks of combination therapy in patients with DC and patients with PD. DC, disease control; Lym, lymphocyte; NK, Natural Killer; PD, progressive. *<0.05.

The application of PD-1 inhibitors in cancer patients can reactivate functions of immune cells, particularly T cells, restoring their immune function to exert antitumor effects (23). Studies have demonstrated that the early response and proliferation of peripheral blood PD-1^+^ CD8^+^ T cells can predict the response to anti-PD-1 therapy in solid tumors (24). In this study, we also conducted an initial assessment of the impact of PD-1 therapy on immune activation in this specific population by analyzing changes in the distribution of lymphocyte subpopulations in patients with HCC combined with hyperbilirubinemia. E (**Figure 3A**). When patients were grouped according to tumor response and changes in lymphocyte subpopulation counts were evaluated among those with different treatment outcomes (**Figures 3B–G**), we observed that both the counts of T cells (*P* =0.047), CD4^+^ T (*P* =0.020), and CD8^+^ T (*P* =0.030) in patients with PD showed progressive decline in T-cell counts. In contrast, these counts remained stable during the 12 weeks of treatment in patients with DC (**Figures 3B, C, G**). The change in NK cell counts between the two groups mirrored the trend observed for T cells, though the difference did not reach statistical significance (**Figure 3E**). These findings suggest that lymphocyte dynamics during PD-1 therapy may correlate with treatment outcomes, warranting further investigation.

### The MELD score and short-term changes of TBIL level are risk factors associated with OS

After 12 weeks of combined PD-1 inhibitors and TKIs treatment, we observed a significant improvement in TBIL levels. However, a slight increase in TBIL was noted within the first week (P=0.026) (**Figure 1A**). This transient rise may be attributed to the progressive worsening of liver function in some patients with compromised hepatic function. Therefore, we conducted a Cox regression analysis to identify prognostic risk factors. To minimize potential bias from the choice of different TKI treatments, we included only patients treated with lenvatinib. After adjusting for age, sex, and other relevant covariates, the multivariate Cox survival analysis revealed that MELD score >18 (*P* =0.016, HR=3.982 [95% CI: 1.292–12.273]) and decrease in TBIL level within the first week(*P* =0.001, HR=0.243 [95% CI: 0.102–0.575]) were significant risk factors impacting OS, as shown in **Table 4**.

**Table 4:** Cox univariate and multivariate survival analysis to identify any risk factor associated with OS^†^.

	Cox univariate survival analysis^‡^				Cox multivariate survival analysis^§^			
	HR	95% CI		*P* value	HR	95% CI		*P* value
Age	0.988	0.961	1.017	0.413	0.991	0.955	1.029	0.652
Sex	0.710	0.252	2.003	0.518	1.468	0.460	4.687	0.517
The occurrence of irAEs	0.864	0.455	1.641	0.656				
TACE treatment	0.585	0.229	1.496	0.263				
PTCD treatment	0.845	0.445	1.606	0.608				
Category of tumor	1.235	0.698	2.183	0.469				
Immunotherapy	1.639	0.630	4.267	0.311				
Line of treatment ^a^	0.484	0.228	1.028	0.059	0.404	0.131	1.247	0.115
BCLC stage	1.029	0.614	1.723	0.914				
Child-Pugh stage ^b^	2.599	1.285	5.255	0.008	1.291	0.463	3.603	0.625
MELD >18	1.629	0.717	3.699	0.244	3.982	1.292	12.273	0.016
Baseline level of AFP≥ULN	1.037	0.529	2.033	0.915				
Baseline level of ALB	0.972	0.917	1.030	0.334				
Baseline level of TBIL	1.001	0.998	1.003	0.645				
Baseline level of TBIL≥10ULN	1.313	0.691	2.497	0.406				
Degraded TBIL ^c^	0.333	0.160	0.693	0.003	0.243	0.102	0.575	0.001
Baseline level of DBIL/TBIL	2.729	0.235	31.751	0.423				
Baseline level of ALT	1.005	1.000	1.011	0.059	1.002	0.997	1.008	0.419
Baseline level of AST	1.001	0.999	1.004	0.308				
Baseline level of ALT/AST	1.090	0.919	1.293	0.321				
Baseline level of ALP	1.000	0.999	1.001	0.942				
Baseline level of γGGT	1.000	0.999	1.001	0.624				
Baseline level of CHE	1.000	1.000	1.000	0.514				
Baseline level of CHE≥ULN	0.806	0.421	1.546	0.517				
Baseline level of CRP	1.004	0.995	1.013	0.357				
Baseline level of CRP≥ULN	2.919	1.029	8.279	0.044	2.057	0.688	6.151	0.197
Baseline level of CRP≥2ULN	1.354	0.697	2.629	0.371				
Degraded CRP ^d^	0.936	0.428	2.048	0.869				
Baseline level of IL-6	1.004	0.997	1.011	0.289				
Elevated IL-6 ^e^	1.143	0.517	2.527	0.742				

^†^All patients included in the analysis received the same TKI agent, lenvatinib. ^‡^Risk factors with *P <*0.1 in the univariate analysis and MELD were included in the multivariate analysis. ^§^Adjusted results were adjusted for age and sex. ^a^ First-line vs. non-first-line treatment. ^b^ Stage A and B vs stage C. ^C^ Level of TBIL at baseline vs level of TBIL at one week. ^d^ Level of CRP at baseline vs level of CRP at one week. ^e^ Level of IL-6 at baseline vs level of IL-6 at one week.

AFP, alpha fetoprotein; ALB, albumin; ALP, alkaline phosphatase; ALT, alanine aminotransferase; AST, aspartate aminotransferase; BCLC, barcelona clinic liver cancer; CHE, cholinesterase; CRP, C-reactive protein; DBIL, direct bilirubin; γGGT, gamma-glutamyl transferase; HR, hazard ratio; IL-6, interleukin-6; irAEs, immune-related adverse events; MELD, model for end-stage liver disease; PTCD, percutaneous transhepatic cholangio drainage; TACE, transcatheter arterial chemoembolization; TBIL, total bilirubin; TKI, tyrosine kinase inhibitor; ULN, upper limit of normal value.

### Patients who exhibited an early decrease in TBIL levels showed longer survival times

**Figure 4:**
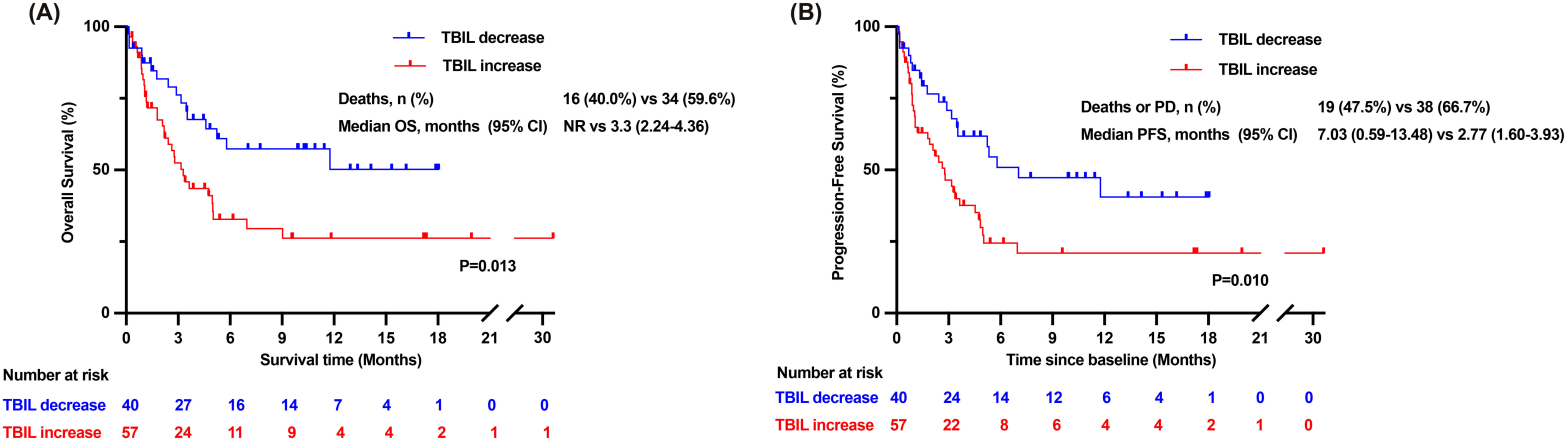
Overall survival time and progression-free survival time of patients in the TBIL-increasing group and TBIL-decreasing group. **(A)** Kaplan-Meier curves of overall survival time for patients in the TBIL-increasing group and the TBIL-decreasing group. **(B)** Kaplan-Meier curves of progression-free survival time for patients in the TBIL-increasing group and the TBIL-decreasing group. CI, confidence interval; NR, Not reached; OS, overall survival; PD, progressive disease; PFS, progression-free survival; TBIL, total bilirubin.

We stratified patients according to the risk factors to assess differences in survival outcomes. Patients who experienced a decrease in TBIL levels within the first week showed a prolonged mOS (not reached [NR] vs 3.3months [95% CI: 2.24–4.36 months], *P* =0.013) and mPFS (7.03 months [95% CI: 0.59–13.48 months] vs 2.77 months [95% CI: 1.60–3.93 months], *P* =0.010) compared to those of patients with TBIL levels consistently elevated during the same period (**Figures 4A, B**).In contrast, patients with a MELD score ≤18 did not show a significantly longer mOS compared to those with a MELD score >18(5.03 months vs 5.52 months, *P* =0.509), as well as no significant difference in mPFS (4.43 months vs 3.53 months, *P* =0.820) (**Supplementary Figures S2A, B**).

To evaluate the impact of different levels of TBIL on PD-1 treatment, we also included a subset of patients with TBIL levels between ULN and 3×ULN and compared the survival outcomes between the two groups. Baseline characteristics are shown in **Supplementary Table S1**. We found that patients with TBIL levels > 3×ULN did not exhibit significant differences in OS or PFS compared to those with lower levels (**Supplementary Figures S2C, D**). The findings suggest that patients with hyperbilirubinemia for whom PD-1 is not recommended by guidelines or drug descriptions have a favorable safety profile when treated with PD-1 plus TKIs combination therapy. This particular group can achieve survival benefits similar to other patients recommended for treatment.

We also grouped the influencing factors to compare the differences in immune cell counts. However, no statistically significant difference was found between the two groups in terms of MELD score (**Supplementary Figure S3**) or early decline in TBIL (**Supplementary Figure S4**).

## Discussion

The progress of PD-1 inhibitors plus TKIs combination therapy in specific subgroups of patients with advanced HCC has been limited due to potential adverse events, liver toxicity, and the possibility of liver failure associated with combination therapy (25). No studies have specifically investidated the efficacy and safety of these combination therapies in patients with more advanced liver injury (Child–Pugh class C or TBIL level >3×ULN) (7, 26, 27). However, there is insufficient evidence that specific populations with hepatic impairment cannot receive combination therapy. IPSOS reported that the PD-L1 inhibitor, atezolizumab, showed significantly improved efficacy over single-agent chemotherapy when used as first-line treatment for patients with NSCLC, who were not suitable for platinum-containing two-agent chemotherapy due to physical status score 2–3 or comorbidities and contraindications to chemotherapy (28). Meanwhile, the application of PD-1 inhibitors plus TKIs combination therapy in pediatric patients with advanced or recurrent malignancies has recently been reported, demonstrating a favorable tolerability and potential efficacy (29). The encouraging findings also provide an important reference for the use of combination therapy in patients with HCC combined with hyperbilirubinemia.

To our knowledge, our study was the first to attempt PD-1 inhibitors combination therapy in patients with HCC combined with hyperbilirubinemia or severe hepatic impairment that achieved surprising outcomes. Tolerability was an important concern in our study. Overall, AEs were manageable for all ICIs combined with TKI treatment, which was consistent with findings in previously reported studies (22, 30). Neither new nor unexpected AEs were observed.

We observed disease control in 61.2% of patients and objective response in 11.9% of patients, accompanied by a trend towards control or improvement of liver function. Interestingly, the patients presented with high TBIL levels at baseline but maintained over 50% PTA at baseline; hence, we considered the presence of simple cholestasis rather than a combination of severe liver failure in such patients (13, 31). In previous reports, patients with HCC combined with hyperbilirubinemia or severe hepatic impairment survived no longer than three months due to poor underlying liver function (1). The meta-analysis results showed that the combination of PD-1/PD-L1 inhibitors with TKIs significantly improved ORR (OR 3.17, P < 0.001), DCR (OR 2.44, P < 0.001), PFS (HR 0.58, P < 0.001), and OS (HR 0.58, P < 0.001) in HCC patients, which is consistent with the results observed in our study (32). The ORR and DCR observed in our study were lower than those reported in previous real-world studies (33–35), but were comparable to the results from similar exploratory studies on expanded indications (36). This is likely related to the poorer baseline liver function of the enrolled patients, who are often excluded from most studies (37, 38). Despite the advanced stage of the tumor and the severe impairment of liver function, this group of patients with advanced tumors can still proceed with treatment attempts and have the potential to clinical benefit from combination therapy. In the HCC microenvironment, impaired CD8^+^ T cells activity were commonly observed, revealing associations with biomarkers and pathways of T cell exhaustion (39). In our study, we observed an increase in the counts of T cells, mainly CD8^+^ T cells, in disease-controlled patients, thus suggesting that some of our patients with hyperbilirubinemia can still respond to PD-1 inhibitors. It also further suggested that blocking PD-1/PD-L1 binding partially restores the function of CD8^+^ T cell to achieve tumor killing, although we did not explore functional changes in CD8^+^ T cells (40, 41). Subsequent and more intensive findings need to be validated, with more samples and studies of the underlying mechanism.

We found that the MELD score and change in TBIL levels within a week were risk factors for OS, where patients with decreased TBIL levels within a week showed a significantly prolonged mOS. Lin et al. demonstrated that patients with advanced HCC could undergo immunotherapy with favorable prognoses after sufficient reduction of bilirubin levels (14). However, for patients who continue to exhibit uncorrectable hyperbilirubinemia after treatment, current guidelines still advise against PD-1 therapy to prevent the risk of worsening potential liver failure. Our study confirmed that these patients, whose TBIL levels cannot be improved, can still safely receive combination therapy. Compared to adding immunotherapy after other treatments, implementing active TBIL-lowering measures while initiating PD-1 combined with TKIs therapy may be more beneficial in prolonging patient survival (42). Therefore, we believe that aggressive therapies to decrease TBIL levels, such as bilirubin adsorption and PTCD used in previous reports, should be adopted at an early stage during treatment with PD-1 inhibitors combined with TKIs (43–45). We compared the influencing factors into groups to analyze the differences between immune cell counts, however, we did not find significant difference in counts of immune cells. Based on this observation, we speculate that, unlike the results observed in the tumor response grouping, the overall prognosis of patients may be more related to underlying liver function rather than immune cell counts.

This research has limitations. Firstly, this study was a single-center retrospective study in which treatment strategies were personalized based on the preferences of primary care physicians and patients, inevitably introducing selection bias. Second, the study did not explore the impact of diverse PD-1 inhibitor interventions on efficacy, although the multifactorial analysis indicated that the selection of various PD-1 inhibitors did not exert a significant influence on OS. Furthermore, the study encountered constraints in sample size. Larger prospective randomized controlled trials are necessary in the future to demonstrate the potential benefit of applying combination therapy in patients with HCC combined hyperbilirubinemia.

In conclusion, there are no previous studies in the patient population with advanced HCC combined with hyperbilirubinemia (TBIL level >3×ULN) and no recommendations for the use of ICIs in this population. This study provided evidence supporting the safety of PD-1 inhibitors in combination with TKIs in patients with HCC combined with hyperbilirubinemia. Our findings provided important insights for expanding the patient population eligible for PD-1 therapy in advanced HCC. Patients with HCC combined with elevated TBIL levels (>3×ULN) can still benefit from combination therapy over conservative therapy. For this subset of patients, combination therapy should be initiated as soon as possible, ideally before TBIL is fully corrected, to ensure they receive the benefits of treatment at the earliest opportunity.

## Data Availability

The original contributions presented in the study are included in the article/**Supplementary Material**. Further inquiries can be directed to the corresponding authors.

## Supplementary material

Supplementary Figure 1 Flow chart. Abbreviations: PD-1, Programmed death-1; TBIL, total bilirubin; PA, prothrombin activity; ULN, upper limit of normal value.

Supplementary Figure 2 Overall survival and progression-free survival time of patients with different groups. **(A)** Overall survival of patients with MELD scores >18 and MELD scores ≤18. **(B)** Progression-free survival of patients with MELD scores >18 and MELD scores ≤18. **(C)** Overall survival of patients with different TBIL levels. **(D)** Progression-free survival of patients with different TBIL levels. CI, confidence interval; MELD, model for end-stage liver disease; NR, Not reached; OS, overall survival; PFS, progression-free survival; TBIL, total bilirubin; ULN, upper limit of normal value.

Supplementary Figure 3 The changes in lymphocyte subsets after combination therapy. **(A)** Changes in lymphocyte count within 12 weeks of combination therapy inpatients with MELD scores >18 and MELD scores ≤18. **(B)** Changes in T cell count within 12 weeks of combination therapy inpatients with MELD scores >18 and MELD scores ≤18. **(C)** Changes in B cell count within 12 weeks of combination therapy inpatients with MELD scores >18 and MELD scores ≤18. **(D)** Changes in NK cell count within 12 weeks of combination therapy inpatients with MELD scores >18 and MELD scores ≤18. **(E)** Changes in CD4+ T cell count within 12 weeks of combination therapy inpatients with MELD scores >18 and MELD scores ≤18. **(F)** Changes in CD8+ T cell count within 12 weeks of combination therapy inpatients with MELD scores >18 and MELD scores ≤18.

Supplementary Figure 4 The changes in lymphocyte subsets after combination therapy. **(A)** Changes in lymphocyte count within 12 weeks of combination therapy inpatients with different TBIL levels. **(B)** Changes in T cell count within 12 weeks of combination therapy inpatients with different TBIL levels. **(C)** Changes in B cell count within 12 weeks of combination therapy in patients with different TBIL levels. **(D)** Changes in NK cell counts within 12 weeks of combination therapy inpatients with different TBIL levels. **(E)** Changes in CD4+ T cell count within 12 weeks of combination therapy inpatients with different TBIL levels. **(F)** Changes in CD8+ T cell count within 12 weeks of combination therapy inpatients with different TBIL levels.

Supplementary Table 1 Baseline characteristics of patients with level of TBIL > 1xULN. Continuous variables are presented as mean ± SD or median (interquartile ranges). AFP, alpha fetoprotein; BA, Bilirubin adsorption; BCLC, barcelona clinic liver cancer; MELD, model for end-stage liver disease; PA, prothrombin activity; PTCD, percutaneous transhepatic cholangio drainage; TACE, transcatheter arterial chemoembolization TBIL, total bilirubin; TKI, tyrosine kinase inhibitor.
