# Supplementary figures and images for: Safety and efficacy of PD-1 inhibitors plus tyrosine kinase inhibitors combination therapy in patients with advanced hepatocellular carcinoma combined with hyperbilirubinemia: a retrospective cohort study
Source: Front Immunol. 2025 Mar 11;16:1530477. doi: 10.3389/fimmu.2025.1530477 (PMC11932989; doi:10.3389/fimmu.2025.1530477)

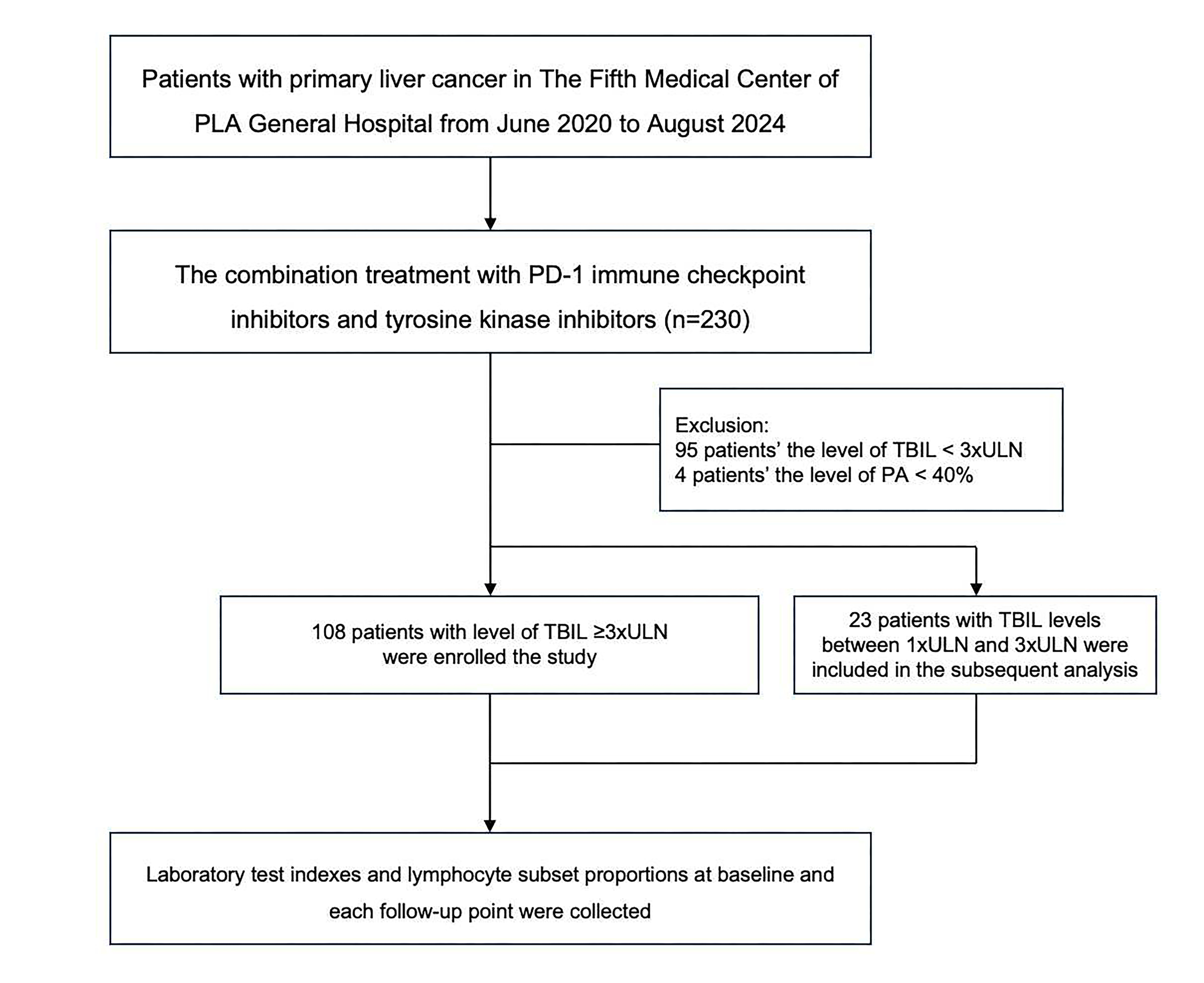

Supplement: Supplementary Figure 1 — Flow chart. Abbreviations: PD-1, Programmed death-1; TBIL, total bilirubin; PA, prothrombin activity; ULN, upper limit of normal value. [file Image1.jpeg]

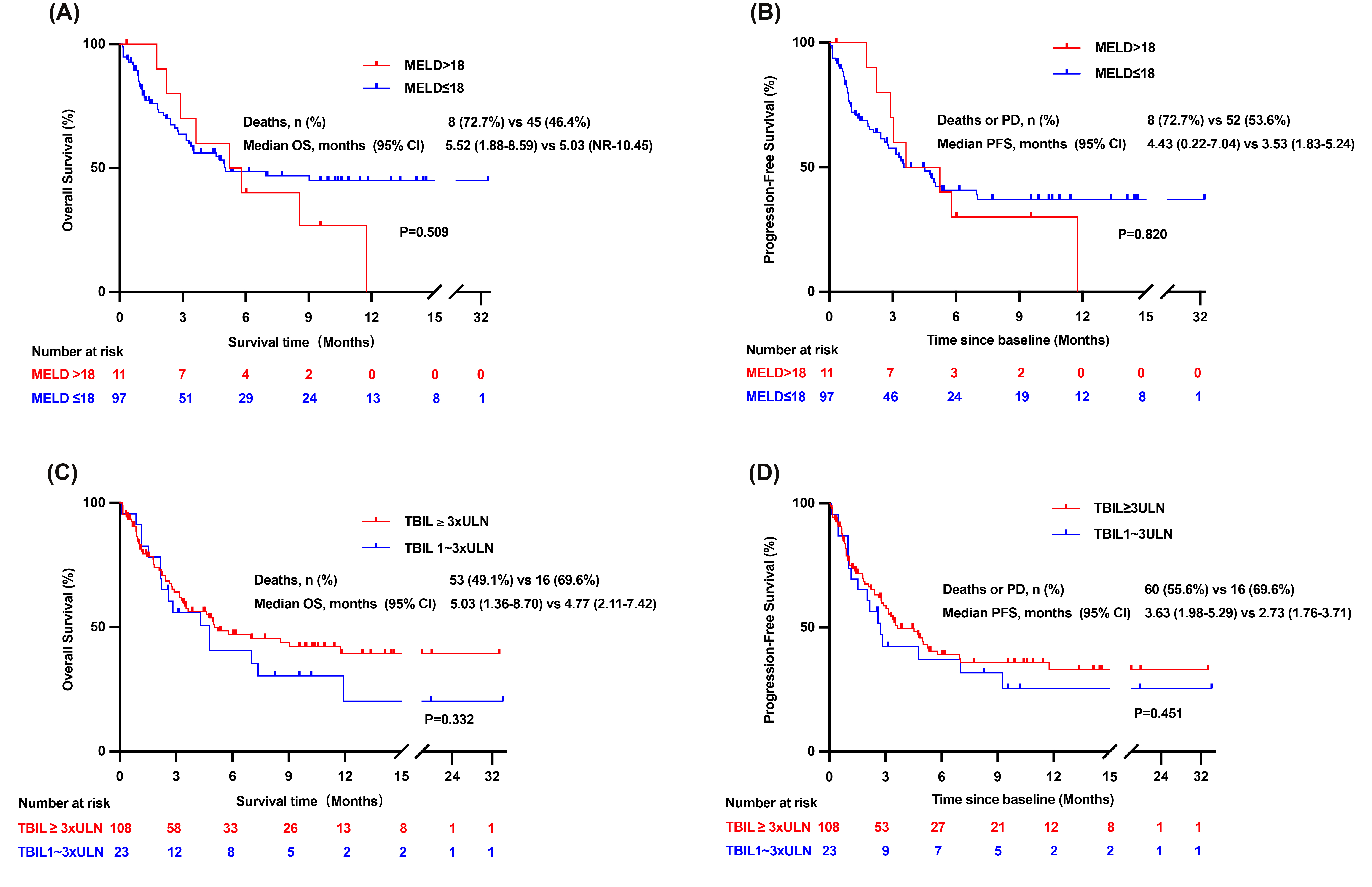

Supplement: Supplementary Figure 2 — Overall survival and progression-free survival time of patients with different groups. (A) Overall survival of patients with MELD scores >18 and MELD scores ≤18. (B) Progression-free survival of patients with MELD scores >18 and MELD scores ≤18. (C) Overall survival of patients with different TBIL levels. (D) Progression-free survival of patients with different TBIL levels. CI, confidence interval; MELD, model for end-stage liver disease; NR, Not reached; OS, overall survival; PFS, progression-free survival; TBIL, total bilirubin; ULN, upper limit of normal value. [file Image2.jpeg]

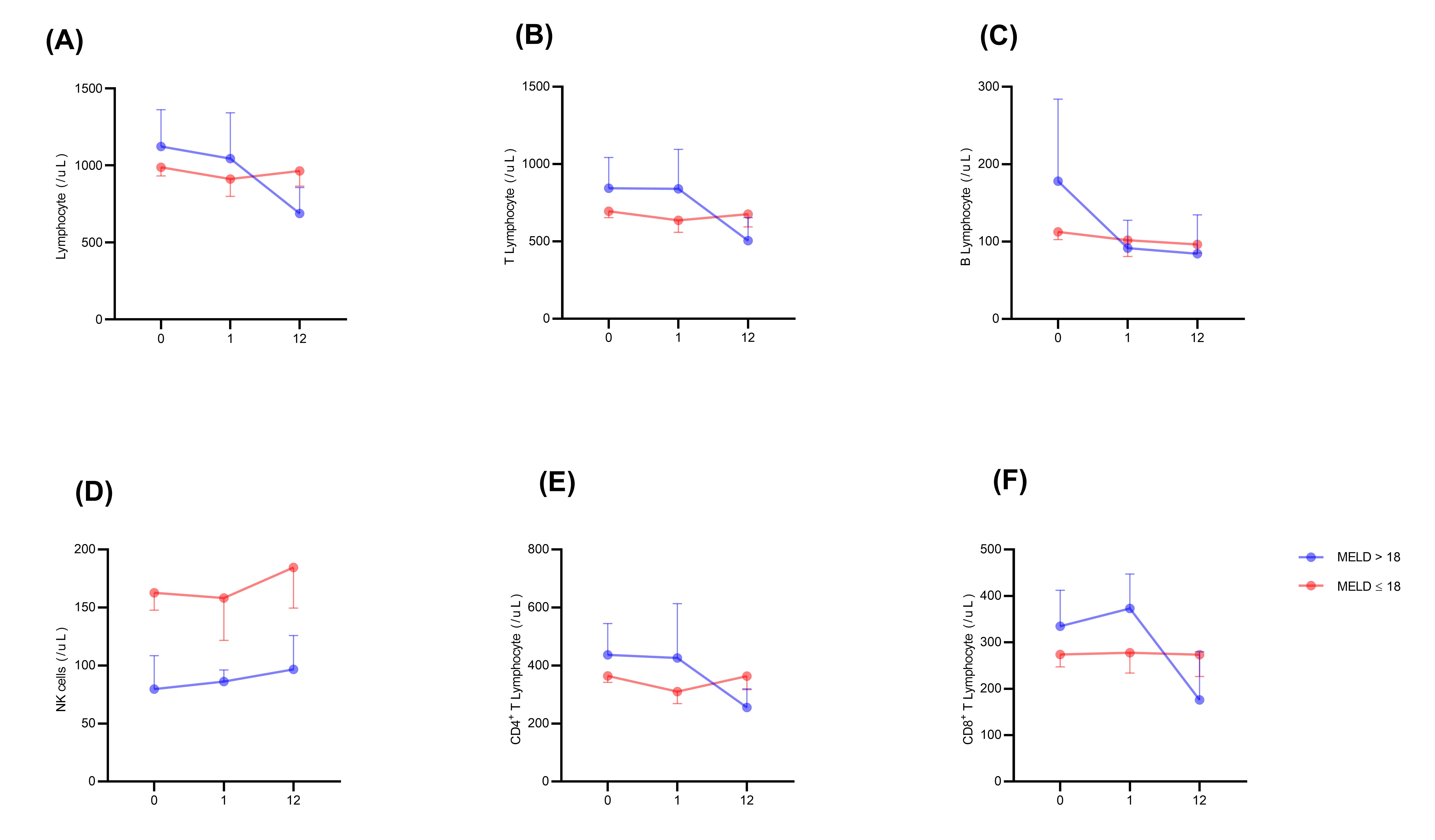

Supplement: Supplementary Figure 3 — The changes in lymphocyte subsets after combination therapy. (A) Changes in lymphocyte count within 12 weeks of combination therapy inpatients with MELD scores >18 and MELD scores ≤18. (B) Changes in T cell count within 12 weeks of combination therapy inpatients with MELD scores >18 and MELD scores ≤18. (C) Changes in B cell count within 12 weeks of combination therapy inpatients with MELD scores >18 and MELD scores ≤18. (D) Changes in NK cell count within 12 weeks of combination therapy inpatients with MELD scores >18 and MELD scores ≤18. (E) Changes in CD4+ T cell count within 12 weeks of combination therapy inpatients with MELD scores >18 and MELD scores ≤18. (F) Changes in CD8+ T cell count within 12 weeks of combination therapy inpatients with MELD scores >18 and MELD scores ≤18. [file Image3.jpeg]

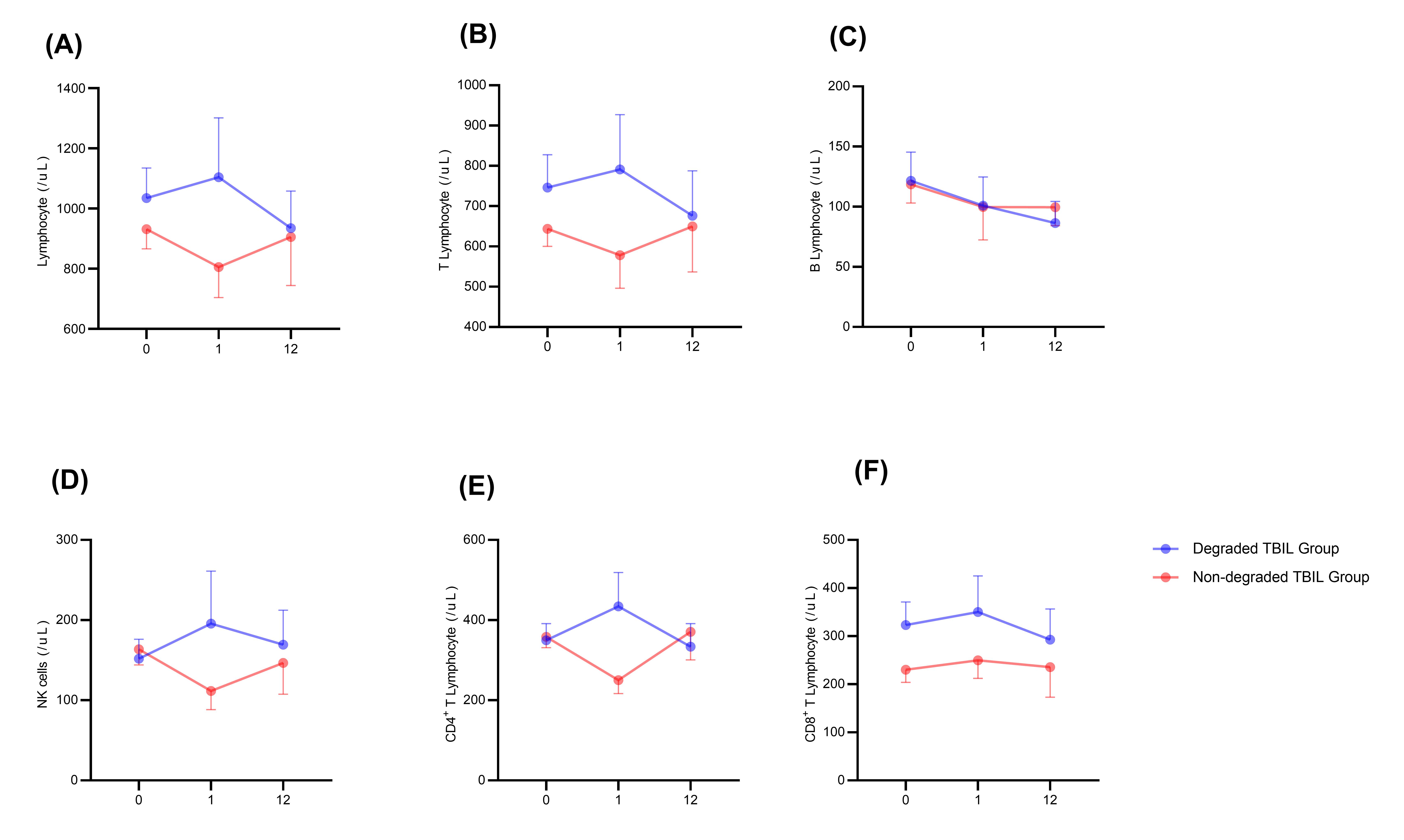

Supplement: Supplementary Figure 4 — The changes in lymphocyte subsets after combination therapy. (A) Changes in lymphocyte count within 12 weeks of combination therapy inpatients with different TBIL levels. (B) Changes in T cell count within 12 weeks of combination therapy inpatients with different TBIL levels. (C) Changes in B cell count within 12 weeks of combination therapy in patients with different TBIL levels. (D) Changes in NK cell counts within 12 weeks of combination therapy inpatients with different TBIL levels. (E) Changes in CD4+ T cell count within 12 weeks of combination therapy inpatients with different TBIL levels. (F) Changes in CD8+ T cell count within 12 weeks of combination therapy inpatients with different TBIL levels. [file Image4.jpeg]
