# Supplementary material for: Safety and efficacy of PD-1 inhibitors plus tyrosine kinase inhibitors combination therapy in patients with advanced hepatocellular carcinoma combined with hyperbilirubinemia: a retrospective cohort study
Source: Front Immunol. 2025 Mar 11;16:1530477. doi: 10.3389/fimmu.2025.1530477 (PMC11932989; doi:10.3389/fimmu.2025.1530477)
Supplement: Supplementary Table 1 — Baseline characteristics of patients with level of TBIL > 1xULN. Continuous variables are presented as mean ± SD or median (interquartile ranges). AFP, alpha fetoprotein; BA, Bilirubin adsorption; BCLC, barcelona clinic liver cancer; MELD, model for end-stage liver disease; PA, prothrombin activity; PTCD, percutaneous transhepatic cholangio drainage; TACE, transcatheter arterial chemoembolization TBIL, total bilirubin; TKI, tyrosine kinase inhibitor. [file Table1.docx]

**TABLE S1 Baseline characteristics of patients with level of TBIL > 1xULN.**

|  | **TBIL ≥ 3xULN**  **n=108 (%)** | **1xULN < TBIL < 3xULN**  **n=23 (%)** | ***P*** |
| --- | --- | --- | --- |
| Age (year) | 55.81±10.19 | 57.83±11.11 | 0.399 |
| Sex |  |  | 0.372 |
| Male | 95(88.6%) | 18(78.3%) |  |
| Female | 13(11.4%) | 5(21.7%) |  |
| Diagnosis |  |  | 0.003 |
| Hepatocellular carcinoma | 74(68.5%) | 21(91.3%) |  |
| Cholangiocarcinoma | 33(30.6%) | 1(4.3%) |  |
| Other | 1(0.9%) | 1(4.3%) |  |
| Basics of hepatitis |  |  | 0.082 |
| Viral hepatitis B | 65(60.2%) | 21(91.3%) |  |
| Viral hepatitis C | 6(5.6%) | 0(0%) |  |
| Alcoholic hepatitis | 15(13.9%) | 1(4.3%) |  |
| Without basics of hepatitis | 21(19.4%) | 1(4.3%) |  |
| Other | 1(0.9%) | 0(0%) |  |
| BCLC stage |  |  | 0.015 |
| A | 2(2.7%) | 0(0%) |  |
| B | 4(5.3%) | 4(18.1%) |  |
| C (PVTT) | 20(26.7%) | 7(31.8%) |  |
| C (M) | 26(34.7%) | 11(50.0%) |  |
| D | 23(30.7%) | 0(0%) |  |
| Tumor node metastasis classification |  |  | 0.353 |
| III | 11(33.3%) | 1(100%) |  |
| IV | 22(66.7%) | 0(0%) |  |
| Child-Pugh stage |  |  | 0.008 |
| A | 5(4.6%) | 4(17.4%) |  |
| B | 80(74.1%) | 19(82.6%) |  |
| C | 23(21.3%) | 0(0%) |  |
| mALBI |  |  | 0.016 |
| 1 | 5(4.6%) | 1(4.3%) |  |
| 2a | 4(3.7%) | 3(13.0%) |  |
| 2b | 39(36.1%) | 14(60.9%) |  |
| 3 | 60(55.6%) | 5(21.7%) |  |
| AFP |  |  | 0.673 |
| <400 ng/ml | 65(60.2%) | 13(56.5%) |  |
| ≥400 ng/ml | 41(38.0%) | 10(43.5%) |  |
| Not available | 2(1.9%) | 0(0%) |  |
| CA19-9 |  |  | 0.448 |
| ≤37 U/ml | 23(21.3%) | 6(26.1%) |  |
| >37 and <1000 U/ml | 64(59.3%) | 10(43.5%) |  |
| ≥1000 U/ml | 10(9.3%) | 2(8.7%) |  |
| Not available | 11(10.2%) | 5(21.7%) |  |
| Baseline of TBIL (μmol/L) | 105.50(65.03, 199.60) | 42.10(35.50, 46.10) | <0.001 |
| Baseline of PA (%) | 68.60(56.90, 80.90) | 64.10(52.50, 88.50) | 0.906 |
| MELD | 11.66±5.39 | 9.57±2.48 | 0.071 |
| Previous antiviral therapy |  |  | 0.546 |
| Yes | 65(60.2%) | 16(69.6%) |  |
| No | 43(39.8%) | 7(30.4%) |  |
| Immune-related adverse events |  |  | 0.483 |
| Yes | 39(36.1%) | 13(56.5%) |  |
| No | 69(63.9%) | 10(43.5%) |  |
| Immunotherapy |  |  | 0.328 |
| Sintilimab | 60(56.1%) | 17(73.9%) |  |
| Camrelizumab | 7(6.5%) | 3(13.0%) |  |
| Tislelizumab | 7(6.5%) | 1(4.3%) |  |
| Cadonilimab | 28(26.2%) | 2(8.7%) |  |
| Toripalimab | 4(3.7%) | 0(0%) |  |
| Durvalumab | 1(0.9%) | 0(0%) |  |
| Combination TKI treatment |  |  | 0.303 |
| Lenvatinib | 86(79.6%) | 20(87.0%) |  |
| Bevacizumab | 5(4.6%) | 1(4.3%) |  |
| Sorafenib | 3(2.8%) | 2(8.7%) |  |
| Rigotinib | 13(12.0%) | 0(0%) |  |
| Apatinib | 1(0.9%) | 0(0%) |  |
| Combinated TACE therapy |  |  | ＞0.999 |
| Yes | 27(25.0%) | 6(26.1%) |  |
| No | 81(75.0%) | 17(73.9%) |  |
| Combinated BA therapy |  |  | 0.353 |
| Yes | 7(6.5%) | 0(0%) |  |
| No | 101(93.5%) | 23(100%) |  |
| Combinated PTCD therapy |  |  | 0.007 |
| Yes(before/after combinational therapy) | [38(30/8)](35.2%) | [1(0/1)](4.3%) |  |
| No | 70(64.8%) | 22(95.7%) |  |
| Lines of systemic therapy |  |  | 0.036 |
| First line | 80(74.1%) | 12(52.2%) |  |
| Second line | 24(22.2%) | 7(30.4%) |  |
| Later line | 4(3.7%) | 4(17.4%) |  |

Continuous variables are presented as mean ± SD or median (interquartile ranges). **Abbreviations**: **AFP**, alpha fetoprotein; **BA**, Bilirubin adsorption; **BCLC**, barcelona clinic liver cancer; **MELD**, model for end-stage liver disease; **PA**, prothrombin activity; **PTCD**, percutaneous transhepatic cholangio drainage; **TACE,** transcatheter arterial chemoembolization **TBIL**, total bilirubin**; TKI**, tyrosine kinase inhibitor.
